# Provider and clinic-level correlates of deferring antiretroviral therapy for people who inject drugs: a survey of North American HIV providers

**DOI:** 10.1186/1758-2652-15-10

**Published:** 2012-02-23

**Authors:** Ryan P Westergaard, Bridget K Ambrose, Shruti H Mehta, Gregory D Kirk

**Affiliations:** 1 Department of Medicine, University of Wisconsin School of Medicine and Public Health, 600 Highland Ave, Madison, WI, USA; 2 Department of Epidemiology, Johns Hopkins Bloomberg School of Public Health, 615 N. Wolfe St, Baltimore, MD, USA; 3 Department of Medicine, Johns Hopkins University, 600 N. Wolfe St, Baltimore, MD, USA; 4 Department of Medicine, University of Wisconsin School of Medicine and Public Health, 1685 Highland Ave, MFCB 5220, Madison, WI 53711-2281, USA

## Abstract

**Background:**

Injection drug users (IDUs) face numerous obstacles to receiving optimal HIV care, and have been shown to underutilize antiretroviral therapy (ART). We sought to estimate the degree to which providers of HIV care defer initiation of ART because of injection drug use and to identify clinic and provider-level factors associated with resistance to prescribing ART to IDUs.

**Methods:**

We administered an Internet-based survey to 662 regular prescribers of ART in the United States and Canada. Questionnaire items assessed characteristics of providers' personal demographics and training, site of clinical practice and attitudes about drug use. Respondents then rated whether they would likely prescribe or defer ART for hypothetical patients in a series of scenarios involving varying levels of drug use and HIV disease stage.

**Results:**

Survey responses were received from 43% of providers invited by email and direct mail, and 8.5% of providers invited by direct mail only. Overall, 24.2% of providers reported that they would defer ART for an HIV-infected patient with a CD4+ cell count of 200 cells/mm^3 ^if the patient actively injected drugs, and 52.4% would defer ART if the patient injected daily. Physicians were more likely than non-physician providers to defer ART if a patient injected drugs (adjusted odds ratio 2.6, 95% CI 1.4-4.9). Other predictors of deferring ART for active IDUs were having fewer years of experience in HIV care, regularly caring for fewer than 20 HIV-infected patients, and working at a clinic serving a population with low prevalence of injection drug use. Likelihood of deferring ART was directly proportional to both CD4+ cell count and increased frequency of injecting.

**Conclusions:**

Many providers of HIV care defer initiation of antiretroviral therapy for patients who inject drugs, even in the setting of advanced immunologic suppression. Providers with more experience of treating HIV, those in high injection drug use prevalence areas and non-physician providers may be more willing to prescribe ART despite on-going injection drug use. Because of limitations, including low response rate and use of a convenience sample, these findings may not be generalizable to all HIV care providers in North America.

## Background

Injection drug use continues to account for a significant proportion of the HIV burden in the United States and Canada [1,2]. In addition to having increased risk of HIV acquisition and transmission, injection drug users (IDUs) tend to have more limited engagement in HIV care and treatment. Data from some clinical and community-based observational cohorts have indicated that active IDUs have inferior virologic [3,4] and immunologic [5,6] responses to antiretroviral therapy (ART) compared with former IDUs and non-drug using patients.

Despite this, we and others have observed survival benefits of ART among IDUs with advanced HIV/AIDS that approaches that observed in other risk groups [7,8]. Studies in other contexts with universal access to ART have demonstrated similar mortality and rates of antiretroviral resistance among IDUs and non-IDUs [9,10], suggesting that the availability of interventions to support adherence and address co-morbid substance abuse may effectively eliminate the ART-related disparities observed in other settings [11].

Mechanisms proposed to mediate the association between injecting drugs and poor HIV treatment outcomes include delayed diagnosis and treatment initiation [12-14], poor retention in outpatient care [15,16], and inadequate medication adherence [5,17]. Additional individual-level correlates of delayed entrance or disengagement from care are older age, black race, and distrust of the medical care system [18,19]. While individual-level and behavioural variables have been the focus of most previous research, some authors have proposed expanding the paradigm used for studying sub-optimal HIV care to encompass social and structural factors, such as stigmatization, drug policies and health care delivery considerations [20,21]. In a nationally representative study, drug-injecting patients whose HIV providers had negative attitudes toward IDUs were significantly less likely to receive ART [22]. IDUs who have favourable perceptions of the relationship with their HIV providers tend to have more appropriate ART utilization and better virologic response [23,24]. Beyond individual providers, contextual factors that have been associated with improved treatment outcomes include aggregate HIV care experience and clinic site specialization [25-27].

Whether underutilization of ART by IDUs is driven to a greater extent by providers' decisions to not recommend treatment or by patients' refusal is unclear, although both scenarios are known to occur [28]. The practice of withholding ART solely on the basis of injection drug use runs counter to clinical guidelines issued by the World Health Organization, which explicitly state that drug injecting should not disqualify patients from ART eligibility, and that addiction treatment should not be required before ART initiation [29]. We investigated this issue by evaluating North American HIV providers' willingness to initiate ART in the context of active injection drug use by their patients. Toward the goal of informing future provider-level interventions to reduce health disparities for HIV-infected drug users, we sought to identify characteristics that distinguish providers who are likely to defer for ART to medically eligible, active IDUs from those who would be likely to prescribe ART despite on-going injecting.

### Participant recruitment

Between October 2009 and May 2010, we recruited health care practitioners who self-identified as regular providers of HIV care to complete an anonymous, 120-item, Internet-based survey. Because one aim of this study was to investigate barriers to optimal HIV care for a cohort of injection drug users in Baltimore, Maryland, USA, we specifically targeted HIV providers in the Baltimore metropolitan area who were identified through local provider databases and clinic staff directories. These Baltimore-based HIV providers were individually invited to participate with up to two mailed letters and three reminders via email, which contained an Internet address and password needed to access the survey.

The sampling frame was then broadened to include providers throughout North America, who were recruited using three complementary approaches: 1) a database of antiretroviral medication prescribers in the state of Maryland and the Washington D.C. metropolitan area identified through the American Medical Association Masterfile; 2) venue-based recruitment at national and international HIV conferences; and 3) an email list of subscribers to an electronic HIV clinical care resource. Providers in Maryland and the Washington D.C. area were sent mailed invitations with instructions for accessing the web-based survey as email addresses were not available.

Venue-based sampling occurred at: annual meetings of the Infectious Diseases Society of America in Philadelphia, PA; the International Antiviral Society-USA in New York, NY; and a clinical HIV care conference hosted by the Johns Hopkins HIV Service in Baltimore. An advertisement inviting eligible providers to complete the survey was circulated to subscribers of an electronic HIV clinical care guide, who could access the survey through a link contained in an email message. Surveys were created and administered using SurveyMonkey (Portland, Oregon). The Johns Hopkins Bloomberg School of Public Health Institutional Review Board reviewed the study protocol and granted it exempt status.

### Response rate

Of 368 providers practicing in Baltimore who were individually invited to participate, 157 (43%) completed surveys. Basic demographic data were available for 168 (80%) of the 211 Baltimore-based non-responders. Compared with participating providers, non-responders were more likely to be male (66% vs. 44%, *p *< 0.001) and have an MD or DO degree (92% vs. 78%, *p *= 0.002) and were less likely to be trained in infectious diseases (8% vs. 54%, *p *< 0.001). Non-Baltimore providers contacted by mail had a response rate of 8.5%. As convenience sampling was utilized to recruit survey participants at professional conferences and via the online list-serve, we could not ascertain response rates relevant to the broader recruitment strategies.

Compared with Baltimore-based providers, those recruited via conferences and the HIV list-serve were more likely to be male (57% vs. 40%, *p *< 0.001), to have specialty training in infectious diseases (76% vs. 56%, *p *< 0.001), to care for 50 or more HIV-infected participants (58% vs. 44%, *p *< 0.001), and were less likely to work at a clinic serving a patient population with a high lifetime prevalence (> 25%) of injection drug use (6% vs. 26%, *p *< 0.001)

### Survey design

This cross-sectional survey assessed demographics and professional characteristics, such as degree type, specialization, years of clinical experience and volume of patient care activities. A series of questions addressed characteristics of the provider's primary practice site, including its geographic location (urban vs. suburban vs. rural), whether it provides mostly primary care or specialty care, and whether it is affiliated with an academic institution. Providers were asked to estimate the proportion of patients seen in their practices who were HIV-infected, had used injection drugs, were underrepresented minorities, and lacked health insurance. Other questions addressed the availability of certain services at the primary practice site, including HIV testing, social work services, substance abuse treatment and transportation assistance.

Provider knowledge, attitudes and beliefs related to injection drug use were assessed based on the degree to which the respondent agreed or disagreed (using a 5-point Likert scale) with a series of statements designed to assess negative or prejudicial attitudes toward IDUs. The statements were drafted by a committee of HIV clinicians and researchers and pilot tested on 15 providers who treat patients in an urban, university-affiliated HIV clinic. Revision and refinement resulted in nine items incorporated into the final survey. Examples include: "I feel uncomfortable talking to my patients about their injection drug use practices"; and "HIV-infected IDUs have themselves to blame for their illness". The full text of all questionnaire items is available in the Additional file 1.

Deferral of ART was assessed by asking respondents to self-rate the likelihood of prescribing ART to a series of hypothetical patients. The range of patient scenarios included a patient with no drug use history, a former IDU who had been abstinent for three months, a current IDU who injects several times per month, and a daily injector. Within each of these four categories of drug use, respondents were further asked to rate the likelihood of deferring ART if the patient in question had a CD4+ cell count of 200 cells/mm^3^, 350 cells/mm^3 ^or 500 cells/mm^3^.

### Statistical analysis

Descriptive statistics related to demographic, provider and clinic characteristics were analyzed using Chi-squared and Fisher's exact tests for categorical data. After sensitivity analyses of potential categorizations, Likert scale variables related to provider attitudes and beliefs were categorized into "agree" (strongly agree and agree) and "do not agree" (neutral, disagree or strongly disagree). Attitude and belief items that showed significant bivariate association with deferral of ART (*p *value for Chi-squared test less than 0.10) were included in subsequent analyses.

The main, binary outcome of interest in this study was a provider report that one would probably defer ART for patient with a CD4+ cell count equal to 200 cell/mm^3 ^if he or she reported any active (i.e., daily or occasional) injection drug use. This approach was chosen because there is near-universal consensus that ART initiation is indicated for patients with this degree of immunosuppression, regardless of symptoms, in both resource-rich and resource-limited settings [30,31]. A decision to defer ART in this context, therefore, likely indicates that a provider believes active injecting precludes effective ART. Logistic regression models were constructed using forward and backward stepwise approaches to identify variables that were independently associated with deferring ART in the setting of any active IDU. Because revised guidelines regarding when to start antiretroviral therapy were published by the US Department of Health and Human Services (DHHS) during the study period [32], we included a covariate indicating whether respondents completed the survey prior to or after 1 December 2009, when the guidelines were released.

In secondary analyses, we repeated these model-building steps for the separate outcomes of ART deferral for drug injecting patients with CD4+ cell counts of 350 cells/mm^3 ^and 500 cells/mm^3^. We next compared the proportion of providers who would defer ART within each of the three strata of CD4+ cell count and the four strata of drug-injecting status, allowing examination of prescribing behaviour across 12 scenarios characterized by varying drug use and disease severity. Finally, we used these data to characterize providers whose decision to defer differed depending on intensity of injecting (i.e., daily vs. occasionally). Statistical analyses were performed using STATA Version 11 (StataCorp, College Station, TX).

## Results

### Characteristics of the study population

Overall, 662 providers from North America responded to the survey: 94.7% reported practicing in the US and 5.3% were from Canada. US-based respondents practiced in 39 different states, with the greatest number coming from Maryland (26%), New York (12%) and Pennsylvania (7%). Demographic, professional and clinical site characteristics are displayed in Table 1. The median age was 43 (IQR 35-52) and the median number of years providing HIV care was 10 years (IQR 4-18).

**Table 1:** Provider and practice site characteristics (n = 662)

Provider characteristics	No. (%)	Clinical site characteristics	No. (%)
Degree		Practice location	

RN/NP/PA	126 (20.5)	Urban	497 (76.6)

MD/DO	488 (79.5)	Suburban	115 (17.7)

Gender		Rural	37 (5.7)

Female	321 (48.8)	Referral level	

Male	337 (51.2)	Primary care	149 (23.0)

Age		Specialty care	279 (43.0)

< 35	134 (20.2)	Hospital based	221 (34.0)

35-50	307 (46.4)	Academic affiliation	

50+	230 (33.4)	No	370 (55.9)

Race/ethnicity		Yes	292 (44.1)

White	409 (61.8)	Proportion of patients who are uninsured	

Asian	123 (18.6)	< 10%	231 (35.6)

Hispanic	42 (6.3)	10-25%	153 (23.6)

Black	49 (7.4)	25%-75%	191 (29.5)

Other/decline	39 (6.0)	More than 75%	73 (11.3)

Specialization		Proportion of patients who are IDU	

Infectious diseases	435 (70.7)	< 10%	350 (54.0)

Primary care	122 (19.8)	10-25%	232 (35.8)

Other	58 (9.4)	More than 25%	66 (10.2)

Years caring for HIV pts.		Substance abuse treatment on site	

< 5y	178 (26.9)	No	365 (55.1)

5-10y	147 (22.2)	Yes	297 (44.9)

10-20y	188 (28.4)	Mental health services on site	

> 20y	148 (22.4)	No	227 (34.3)

Number of HIV patients		Yes	435 (65.7)

cared for regularly			

< 20	121 (18.3)	**Recruitment method**	**No. (%)**

20-50	192 (29.0)	Direct mail and/or email	184 (27.8)

50-100	117 (17.7)	HIV conferences	251 (37.9)

> 100	232 (35.0)	HIV list-serve	227 (34.3)

Amount of workload comprised of clinical care			

Less than 25%	141 (21.3)		

About half	117 (17.7)		

More than 75%	404 (61.0)		

Most respondents were physicians (79.5%), had received specialty training in infectious diseases (70.7%), and reported that more than 75% of their professional activity entailed providing direct patient care (61%). The majority of providers (81.7%) reported regularly providing care for at least 20 HIV-infected patients; 35% reported caring for more than 100 HIV-infected patients regularly. Compared with physician respondents, non-physician providers (mostly nurse practitioners and physician assistants) were older (*p *< 0.001), had been practicing longer (*p *= 0.002), and were more likely to regularly care for more than 100 HIV-infected patients (*p *= 0.02) or to practice in a setting with a high density of IDUs (*p *= 0.004).

Most providers characterized their primary practice location as urban (76.6%). Clinic sites were more commonly described as specialty clinics (43%) than primary care (23%) or hospital-based clinics (34%). Fewer than half of providers (44.1%) indicated that their clinic was affiliated with an academic institution. More than half of providers (54%) estimated that the prevalence of injection drug use among patients served by their primary clinic was less than 10%; few respondents (10.2%) reported that their local prevalence of injection drug use exceeded 25%.

Responses to four survey items assessing attitudes and beliefs that showed a significant bivariate association with deferral of ART are listed in Table 2. A majority of providers disagreed or felt neutral about the statements, "Health care providers have little influence over patients' IDU practices" and "I feel uncomfortable talking to my patients about their IDU practices". A small minority of providers agreed with the statement, "Providers are not professionally obligated to care for IDUs with HIV infection". Approximately half of providers agreed with the statement, "Even former IDUs have difficulty adhering to HAART".

**Table 2:** Beliefs and attitudes towards IDUs among survey respondents (n = 662)

Strongly disagree	Disagree	Neutral	Agree	Strongly agree
*"Even former IDUs have difficulty adhering to HAART"*				

66 (10.2)	253 (39.0)	120 (18.5)	186 (28.7)	24 (3.7)

*"Health care providers have little influence over patients' IDU practices"*				

74 (11.4)	341 (52.5)	124 (19.1)	95 (14.6)	15 (2.3)

*"I feel uncomfortable talking to my patients about their IDU practices"*				

258 (39.8)	293 (45.2)	23 (3.5)	53 (8.2)	22 (3.4)

*"Providers are not professionally obligated to care for IDUs with HIV"*				

292 (45.0)	241 (37.1)	68 (10.5)	37 (5.7)	11 (1.7)

### Provider and clinic-level correlates of deferring ART

Table 3 displays unadjusted and adjusted odds ratios describing provider characteristics and clinic-level factors associated with deferring ART for active IDUs with CD4+ counts of 200 cells/mm^3^. Overall, 24.2% of providers would defer ART for a patient who occasionally injected drugs, while 52.4% would defer ART for daily injection drug use. Multivariate analysis indicated that physicians were significantly more likely than non-physician providers to defer ART for active IDUs (adjusted OR 2.6; 95% CI 1.4-4.9). Providers with greater HIV care experience and a larger panel of HIV-infected patients were significantly less likely to defer ART at this level, although providers who reported that greater than 75% of their workload was direct patient care (rather than research, teaching or administration) were significantly more likely to defer ART. Furthermore, the subset of providers having both the highest clinical workload but the fewest number of regular HIV patients (< 20) were particularly likely to defer therapy, with 40% reporting they would defer ART in this setting (data not shown).

**Table 3:** Likelihood of deferring ART for HIV-infected patients who report active injection drug use

	Likely to defer ART at CD4+ 200 cells/mm^3^		
**Provider characteristics**	**No. (%)**	**Unadjusted OR**	Adjusted OR*

Gender			

Female	63 (19.6)	-	

Male	88 (26.11)	1.45 (1.00-2.09)	

Age			

< 35	33 (24.6)	-	

35-50	89 (29.0)	1.25 (0.79-1.98)	

50+	31 (14.1)	0.5 (0.29-0.87)	

Degree			

RN/NP/PA	14 (11.1)	-	-

MD/DO	125 (25.6)	2.75 (1.52-4.98)	2.61 (1.39-4.92)

Infectious disease specialization			

No	41 (22.8)	-	

Yes	101 (23.2)	1.03 (0.68-1.55)	

Years caring for HIV pts.			

Per 5y increase		0.83 (0.74-0.93)	0.84 (0.73-0.95)

Number of HIV patients cared for regularly			

< 20	43 (35.5)	-	-

20-50	45 (23.4)	0.56 (0.34-0.92)	0.59 (0.33-1.05)

50-100	25 (21.4)	0.49 (0.27-0.88)	0.67 (0.35-1.29)

> 100	40 (17.2)	0.38 (0.23-0.63)	0.45 (0.25-0.83)

Amount of workload comprised of clinical care			

less than 25%	24 (17.0)	-	-

about half	22 (18.8)	1.13 (0.60-2.14)	1.24 (0.57-2.73)

more than 75%	107 (26.5)	1.76 (1.07-2.87)	2.45 (1.35-4.45)

**Clinical site characteristics**			

Substance abuse treatment on site			

No	97 (26.6)	-	

Yes	56 (18.9)	0.64 (0.44-0.93)	

Proportion of patients who are IDU			

< 10%	91 (26.2)	-	-

10-25%	48 (20.8)	0.74 (0.50-1.1)	0.83 (0.52-1.32)

More than 25%	8 (12.1)	0.39 (0.18-0.85)	0.41 (0.16-1.03)

**Provider attitudes**			

*"Even former IDUs have difficulty adhering to HAART"*			

Disagree or neutral	101 (30.6)	-	-

Agree	47 (14.7)	0.39 (0.27-0.58)	0.42 (0.28-0.65)

*"Health care providers have little influence over patients' IDU practices"*			

Disagree or neutral	114 (21.1)	-	

Agree	34 (30.9)	1.67 (1.06-2.63)	

*"I feel uncomfortable talking to my patients about their IDU practices"*			

Disagree or neutral	130 (22.6)	-	

Agree	18 (24.0)	1.08 (0.61-1.90)	

*"Providers are not professionally obligated to care for IDUs with HIV"*			

Disagree or neutral	121 (20.1)	-	-

Agree	27 (56.2)	5.1 (2.79-9.33)	4.79 (2.48-9.23)

*Adjusted for all other variables in the model

Compared with low injection drug use prevalence settings, providers practicing at clinics serving communities with high injection drug use prevalence (< 25%) were 60% less likely to defer therapy because of active injecting (OR 0.4; 0.2-1.0). Of the provider attitude and belief question items, agreeing that "Providers are not professionally obligated to care for IDUs with HIV" was associated with a five-fold increased likelihood of deferring ART (OR 5.1; 2.8-9.3). Providers who agreed that "Even former drug users have difficulty adhering to HAART" were 60% less likely to defer ART (OR 0.4; 0.2-1.0) compared with providers who disagreed with or felt neutral about this statement.

Similar but non-identical provider and clinic characteristics were found to be predictive of deferring ART for drug injectors with CD4+ cell levels of 350 cells/mm^3 ^and 500 cells/mm^3^. Considering active IDUs with CD4+ counts of 350 cells/mm^3^, Physicians were again more likely to defer ART (OR 1.9; 1.2-2.9), as were providers who spend more than 75% of their time in clinical care (OR 2.0; 1.3-3.1). Working at a clinical site with substance abuse treatment services available was associated with decreased odds of ART deferral, although no association with community prevalence of injection drug use was observed at the 350 cells/mm^3 ^CD4+ level. In the scenario of a patient with a CD4+ count of 500 cells/mm^3^, the majority of providers (61.5%) indicated that they would defer therapy regardless of the presence of injection drug use. In this setting, physicians were no more likely than non-physician providers to defer ART because of active injecting. Infectious disease-trained physicians were more likely to defer therapy than providers without specialty training (OR 2.2; 1.4-3.46), an association not observed at lower CD4+ counts. Providers who agreed with the statement, "I feel uncomfortable talking to my patients about their IDU practices", were significantly less likely to defer ART for a patient with CD4+ count of 500 cells/mm^3 ^and active injection drug use (OR 0.5; 0.3-1.0).

In sub-group analyses, similar associations of provider characteristics with ART deferral were observed when data were restricted to Baltimore area providers only and to those identified through venue-based or to list-serve recruitment (data not shown). Overall rates of ART deferral differed somewhat according to whether the date of survey completion was before or after publication of the 2009 DHHS Antiretroviral Treatment Guidelines. Comparing respondents after 12 December 2009 to those before this date, smaller proportions of providers would defer ART for active IDUs with CD4+ counts of 350 cells/mm^3 ^(43.4% and 52.2%, respectively, *p *= 0.03) and 500 cells/mm^3 ^(81.9% and 87.5%, *p *= 0.05). No difference in ART deferral was observed according to date of survey response for the CD4+ level of 200 cells/mm^3 ^(22% vs. 25%, *p *= 0.3). Including date of survey response as a covariate did not change the associations described for any of the three multivariate models.

### Influences of CD4+ level and intensity of drug injecting

**Figure 1:**
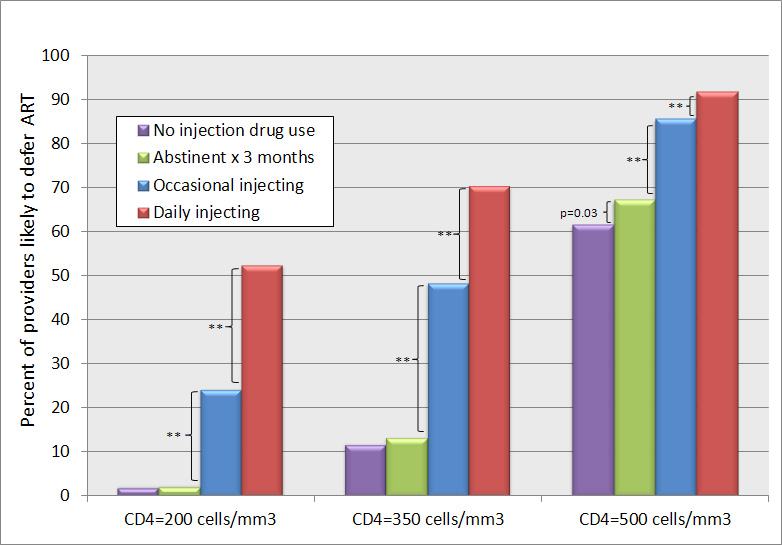
**Percentage of providers who would defer ART by CD4+ count and injection drug use status**.

Figure 1 illustrates the percentage of providers who reported that they would defer ART for patients at three CD4+ levels (200, 350 and 500 cells/mm^3^) and in four different drug use scenarios (no injecting history, former injecting but abstinent for three months, active but less than daily injecting, and daily injecting). Within each of the three CD4+ count strata, we observed a stepwise increase in the proportion likely to defer ART as intensity of injection drug use increased. As expected, virtually all providers would prescribe ART to a typical patient with no substance abuse history and a CD4+ count of 200 cells/mm^3^. Nearly one-fourth of providers (24.2%), however, would defer ART if an otherwise similar patient reported occasional injection drug use, and more than half (52.4%) would withhold therapy if a patient injected daily. Large and statistically significant differences in likely prescribing were observed between the former injecting and occasional injecting scenarios within each CD4+ count category (*p *< 0.001 for each comparison). There were further large differences in likely prescribing practices between the occasional injecting and daily injecting conditions within each CD4+ count category (*p *< 0.001).

Whether providers viewed ART eligibility differently for patients who never injected drugs and former IDUs (in remission for three months) appeared to depend on the degree of immunosuppression. At CD4+ counts of 200 and 350 cells/mm^3^, providers were similarly unlikely to defer ART for patients without a history of injection drug use and for those with former injection drug use who had been abstinent for three months. Providers were, however, slightly more likely to defer therapy for former IDUs than for patients with no history of injection drug use if the CD4+ count was 500 cells/mm^3 ^(67.2% vs. 61.5% would defer ART, *p *= 0.03).

### Discordant prescribing for occasional versus daily IDUs

Next, we aimed to characterize providers who indicated that their decision to prescribe ART to an active IDU would differ depending on the intensity of drug use (daily vs. occasional injecting). While a slight majority of providers (347 of 662, 52%) indicated that they would defer ART for IDUs with daily injection and a CD4+ count of 200 cells/mm^3^, most of these providers (194 of 347, 56%) alternatively reported they would prescribe ART to a similar patient with intermittent injecting. Compared with those who deferred ART with any active injecting, providers willing to prescribe in the setting of occasional injecting were more likely to be non-physician providers, to have greater HIV care experience, to work in an academic setting, and to practice at a site that serves a higher density of IDUs and provides on-site services for substance abuse treatment.

## Discussion

In this cross-sectional survey of providers of regular HIV care, we found that active injection drug use appears to be a major factor in the decision to administer antiretroviral therapy. While virtually all providers studied indicated that they would prescribe ART for a typical patient with CD4+ cell count of 200 cells/mm^3^, nearly one in four providers said that they would defer treatment if a patient reported any injection drug use, and more than half would defer therapy if a patient reported daily injection drug use. Relative differences in ART deferral for drug injectors versus non-injectors were even more pronounced at higher CD4+ counts when initiation of ART is generally considered to be less urgent.

A prominent finding in the multivariate models for the lower CD4+ count categories was that physicians were more likely to defer ART because of injection drug use than non-physician providers. This is in contrast to findings of a previous HIV provider survey by Loughlin *et al*., who found that non-physician providers were more likely than physicians to be resistant to prescribing ART to IDUs [33]. In the prior study, non-physicians had less clinical HIV experience than their physician counterparts, and were more likely to work in a setting with a higher prevalence of drug use. By contrast, the non-physician providers surveyed in the present study had been in practice longer and tended to see a higher volume of HIV-infected patients. Comparison of the two studies may suggest that clinician experience and practice setting, rather than degree type, are important correlates of ART-prescribing behaviour. While the potential for unmeasured confounding exists, adjustment for size of HIV-infected patient panel and injection drug use prevalence did not eliminate the observed association between degree type and likelihood of deferring ART. Further study that incorporates qualitative interviews with providers may lead to better understanding of this discrepancy in view of the complex issues that providers encounter when deciding to initiate ART.

Previous research has indicated that patients who receive care from providers with expertise in HIV medicine are more likely to receive appropriate combination ART [34-37], and in the "pre-HAART era", to have had improved survival [25,26]. In our study, specialty training in infectious diseases was not associated with ART deferral at low CD4+ counts, although those with infectious diseases training were more likely to defer ART at a CD4+ count of 500 cells/mm^3 ^if a patient injected drugs. Having a smaller volume of HIV-infected patients in one's clinic panel was associated with deferring ART for active IDUs. Deferring ART was also more common among providers who provide direct patient care for more than 75% of their professional effort.

Taken together, these findings suggest that busy, full-time clinicians with a relatively small panel of HIV-infected patients may be particularly likely to under-prescribe ART because of injection drug use. In our study, the subset of providers engaged in greater than 75% of patient care but who regularly care for fewer than 20 patients were those most likely to defer ART for IDUs with advanced HIV, with 40% reporting that they would defer ART at a CD4+ count of 200 cells/mm^3^. Our findings are thus in agreement with previous authors who have suggested that provision of regular care to at least 20 to 25 HIV-infected patients is an appropriate minimum standard to be considered to be a qualified HIV provider [38].

Provider judgement about likely adherence is an important factor in the decision to prescribe ART [39]. We correspondingly hypothesized that providers who more strongly believe that IDUs have poor adherence would be more likely to defer ART in the setting of active injecting. Unexpectedly, we found that providers who agreed or strongly agreed with the statement, "Even former IDUs have difficulty adhering to HAART", were significantly less likely to defer ART for IDUs with low CD4+ counts. A possible explanation for this surprising result is that rather than identifying providers who are highly sceptical of the effectiveness of ART for drug users and would be therefore reluctant to recommend it, the survey item distinguished providers who recognize the chronic, relapsing nature of drug dependence. Providers who accept the chronic disease paradigm of drug dependence may have superior knowledge of strategies to manage addiction, and therefore have greater optimism that HIV and substance abuse can be effectively co-managed.

There are several limitations to our study that may restrict its generalizability. We utilized a convenience sampling technique, taking advantage of venues that attract large numbers of HIV providers in order to recruit participants from diverse backgrounds. Providers who agreed to participate are not representative of all attendees of HIV-related conferences, nor are conference attendees and list-serve subscribers representative of all HIV providers who provide care to IDUs in North America. We attempted to mitigate this source of bias by collecting detailed demographic and professional data from participants, and by recruiting a sufficiently large sample size that multivariate analysis could be used to adjust for important confounders.

We expect that over-sampling of providers in the Baltimore-Washington, D.C. area resulted in data collection from multiple providers who work in the same clinic. This clustering of providers may result in correlation of some variables at the clinic level. As the survey did not ask participants to reveal the name or location of their specific clinical sites, we were not able to statistically account for this clustering. Provider self-report is a sub-optimal method of assessing prescribing behaviour and adherence with practice guidelines due to imperfect recall and the possibility of socially desirable reporting [40].

Use of an Internet-based survey instrument has advantages in this regard. An anonymous, self-administered survey which providers can complete in private at a time and place of their choice may minimize this potential bias. As the proportion of personally invited providers who completed surveys was lower in our study than several mail-based surveys of HIV providers [22,33,41], it is possible that web-based questionnaires are a less effective strategy for achieving favourable survey response rates from health care providers than traditional mailings.

## Conclusions

Our analyses suggest that HIV providers are significantly less likely to recommend antiretroviral therapy at any CD4+ cell count for patients who engage in any injection drug use. While many providers would consider prescribing ART to patients who inject occasionally, most would defer therapy for patients who inject daily, even for patients who meet immunologic criteria for AIDS. Providers with greater HIV care experience and non-physician providers may be less resistant to prescribing ART in the setting of injection drug use. Efforts to reduce disparities in ART utilization and HIV treatment effectiveness for IDUs should acknowledge that many providers may be reluctant to prescribe treatment for this marginalized patient group.

## Competing interests

The authors declare that they have no competing interests.

## Authors' contributions

All authors participated in the conception, design and administration of the study. RW and BA performed statistical analyses. RW wrote the initial draft of the manuscript. All authors read and approved the final manuscript.

## Supplementary Material

Additional file 1 **Copy of provider survey**. Click here for file
