# Supplementary material for: Provider and clinic-level correlates of deferring antiretroviral therapy for people who inject drugs: a survey of North American HIV providers
Source: J Int AIDS Soc. 2012 Feb 23;15:10. doi: 10.1186/1758-2652-15-10 (PMC3306203; doi:10.1186/1758-2652-15-10)
Supplement: Additional file 1 — Copy of provider survey. [file 1758-2652-15-10-S1.DOC]

ID___________________ Date Completed_________________

**HIV Provider Survey**

**Demographics (1-3)**

| Do you currently manage the care of patients with HIV disease?*** | Yes  No | *****NOTE:**  If “No”, You are not eligible for this particular survey. We are sorry for any inconvenience and thank you for your time.  If “Yes”, continue. |
| --- | --- | --- |

**Demographics (2-4)**

| How long have you been in practice? | ______­­­_______ years | | How long have you been providing HIV care? | | | | ______­­­_______ years | |
| --- | --- | --- | --- | --- | --- | --- | --- | --- |
| To how many HIV+ patients do you regularly provide care? | Less than 20 | 20-50 | | | 50-100 | | | More than 100 |
| Primary professional activity: | HIV Clinical Care | Other Clinical Care | | | Medical Administration | | | Research |
| What percentage of your professional activity is direct patient contact? | Less than 25% | | | About Half | | More than 75% | | |

**Demographics (3-4)**

| Gender | Male | Female  Decline to Answer | | Age | _________ years |
| --- | --- | --- | --- | --- | --- |
| Race/Ethnicity | White (Non-Hispanic) | | Black/African-American | | Hispanic/Latino |
|  | Asian/Pacific Islander  Decline to Answer | | Other (specify) __________________________ | | |

**Demographics (4-4)**

| Professional Degree | RN | NP | PA | MD | DO |
| --- | --- | --- | --- | --- | --- |
|  | Other (Specify) _________________________ | | | | |
| Please select your specialty  (if applicable) | Family Practice  Infectious Disease  Internal Medicine  OB/GYN  Pediatrics/Adolescent Medicine  Other | | | | |

**Treatment Practices (1-4)**

| **In the next set of questions, we ask about your treatment practices for HIV-infected patients with varying substance abuse behaviors.**  **Please indicate how likely you would be to prescribe HAART to patients with the following CD4 cell counts:** |
| --- |

**Treatment Practices (2-4)**

| **FOR A HYPOTHETICAL PATIENT WITH A CD4 COUNT OF 200 AND…** | **Likely to Prescribe HAART** | **NOT Likely to Prescribe HAART** |
| --- | --- | --- |
| No Substance Abuse |  |  |
| Abuses Alcohol |  |  |
| Abuses Non-Injection Drugs |  |  |
| No Injection Drugs for 3 months |  |  |
| Injects Several Times / month |  |  |
| Injects Daily |  |  |

**Treatment Practices (3-4)**

| **FOR A HYPOTHETICAL PATIENT WITH A CD4 COUNT OF 350 AND…** | **Likely to Prescribe HAART** | **NOT Likely to Prescribe HAART** |
| --- | --- | --- |
| No Substance Abuse |  |  |
| Abuses Alcohol |  |  |
| Abuses Non-Injection Drugs |  |  |
| No Injection Drugs for 3 months |  |  |
| Injects Several Times / month |  |  |
| Injects Daily |  |  |

**Treatment Practices (4-4)**

| **FOR A HYPOTHETICAL PATIENT WITH A CD4 COUNT OF 500 AND…** | **Likely to Prescribe HAART** | **NOT Likely to Prescribe HAART** |
| --- | --- | --- |
| No Substance Abuse |  |  |
| Abuses Alcohol |  |  |
| Abuses Non-Injection Drugs |  |  |
| No Injection Drugs for 3 months |  |  |
| Injects Several Times / month |  |  |
| Injects Daily |  |  |

| How many visits do you typically require from your patients prior to initiating HAART? | 1 visit | 2 visits | 3-4 visits | > 5 visits | Doesn’t matter |
| --- | --- | --- | --- | --- | --- |

**Practice Characteristics (1-1)**

| Do you routinely prescribe methadone for drug treatment? | No  Yes | Do you routinely prescribe buprenorphine for drug treatment? | No  Yes |
| --- | --- | --- | --- |

**Feelings and Beliefs (1-1)**

| **PLEASE INDICATE HOW MUCH YOU AGREE OR DISAGREE WITH THE FOLLOWING STATEMENTS:** | **Strongly Agree** | **Agree** | **Not Sure** | **Disagree** | **Strongly Disagree** |
| --- | --- | --- | --- | --- | --- |
| There is a professional obligation to treat injection drug users (IDUs) with HIV/AIDS. |  |  |  |  |  |
| Many of my HIV infected patients cannot adhere to treatment regimens. |  |  |  |  |  |
| I would not initiate HAART for a patient if they reported active injection drug use. |  |  |  |  |  |
| Even former drug users have difficulty adhering to HAART. |  |  |  |  |  |
| HIV infected IDUs have themselves to blame for their illness. |  |  |  |  |  |
| Health care providers have little influence over their patients’ injection drug use practices. |  |  |  |  |  |
| I feel uncomfortable talking to my patients about their injection drug use practices. |  |  |  |  |  |
| I have a responsibility to talk to my HIV infected patients about safer injection practices. |  |  |  |  |  |
| Needle exchange is effective in reducing HIV transmission. |  |  |  |  |  |

**Practice Site Characteristics** (1-5)

| **The following questions relate to the practice site where you see the majority of your HIV+ patients.**  **Which best describes the practice site where you primarily provide HIV care? Please answer as best you can.** | | | | | | |
| --- | --- | --- | --- | --- | --- | --- |
| Practice Location | Urban | | Suburban | | Rural | |
| Practice Venue | Primary Care | Single-specialty | | Multi-specialty | | Hospital Based |
| Practice Type | Private | Academic | | Public Health | | Other |

**Practice Site Characteristics (2-5)**

| **FOR THE PRACTICE SITE WHERE YOU SEE MOST OF YOUR HIV+ PATIENTS, PLEASE ESTIMATE THE PROPORTION OF HIV+ PATIENTS WHO ARE…** | **0%-10%** | **10%-25%** | **25%-50%** | **50%-75%** | **75%-100%** |
| --- | --- | --- | --- | --- | --- |
| White (non Hispanic) |  |  |  |  |  |
| Black/African-American |  |  |  |  |  |
| Hispanic/Latino |  |  |  |  |  |
| Asian/Pacific Islander |  |  |  |  |  |
| Other Race/Ethnicity |  |  |  |  |  |

**Practice Site Characteristics (3-5)**

| **FOR THAT SAME SITE, PLEASE ESTIMATE THE PROPORTION OF HIV+ PATIENTS WHO ARE…** | **0%-10%** | **10%-25%** | **25%-50%** | **50%-75%** | **75%-100%** |
| --- | --- | --- | --- | --- | --- |
| Currently on HAART |  |  |  |  |  |
| Active IDUs |  |  |  |  |  |
| Former IDUs |  |  |  |  |  |
| Gay, Lesbian, Bisexual, or Transgendered |  |  |  |  |  |

**Practice Site Characteristics (4-5)**

| **WITH RESPECT TO YOUR PAYER MIX, WHAT PERCENT OF YOUR PAYMENTS COME FROM EACH OF THE FOLLOWING SOURCES?** | **0%-10%** | **10%-25%** | **25%-50%** | **50%-75%** | **75%-100%** |
| --- | --- | --- | --- | --- | --- |
| Government (e.g., Medicaid, VA) |  |  |  |  |  |
| Private Insurance |  |  |  |  |  |
| Out-of-Pocket |  |  |  |  |  |
| Uncollected Billables |  |  |  |  |  |

| Does your practice site receive Ryan White Funding? | Yes | No | | Don’t Know |
| --- | --- | --- | --- | --- |
| How long is your average visit… | for new patients? _______ minutes | | for returning patients? _______ minutes | |

**Practice Site Characteristics (5-5)**

| Does your primary practice site offer any of the following services?  (check all that apply) | Substance abuse treatment  HIV testing  Mental health services  Social work services  Childcare | Transportation services (i.e. shuttle)  Transportation reimbursement (i.e.  tokens/voucher)  Pharmacy services  None of the above |
| --- | --- | --- |

**Chronic Disease Screening Practices (1-1)**

| **The next set of questions refers to chronic disease screening practices you may have carried out within the last month. Please remember, this is just for your HIV+ patients. Thank you for your continued participation.**  **You’re almost finished!** | | | | | | |
| --- | --- | --- | --- | --- | --- | --- |
| **DURING THE PRIOR 3 MONTHS, FOR HOW MANY OF YOUR HIV+ PATIENTS DID YOU...** | **ALL HIV+ PATIENTS** | **MOST HIV+ PATIENTS** | **HALF OF HIV+ PATIENTS** | **A FEW HIV+ PATIENTS** | **NONE OF HIV+ PATIENTS** | **N/A** |
| Perform an annual cholesterol test for males over age 35 and females over age 45? |  |  |  |  |  |  |
| Recommend that your male patients over age 50 get colonoscopy screening? |  |  |  |  |  |  |
| Recommend that your female patients over age 40 get a mammogram every  1-2 years? |  |  |  |  |  |  |
| Recommend or perform a Pap smear every 1-3 years for your female patients? |  |  |  |  |  |  |
| Formally screen for major depression? |  |  |  |  |  |  |
| Formally screen for female patients for intimate partner violence? |  |  |  |  |  |  |
| Ask about their tobacco use status, regardless of their smoking history? |  |  |  |  |  |  |
| Advise to quit (of those who reported smoking cigarettes)? |  |  |  |  |  |  |

**Tobacco Use Assessment and Cessation Advice (1-2)**

| **FOR THE NEXT SET OF QUESTIONS, PLEASE INDICATE TO WHAT EXTENT YOU AGREE WITH THE FOLLOWING STATEMENTS:** | **Strongly Agree** | **Agree** | **Not Sure** | **Disagree** | **Strongly Disagree** |
| --- | --- | --- | --- | --- | --- |
| Many of my HIV+ patients have no interest in quitting smoking. |  |  |  |  |  |
| Among all of my other responsibilities, I do not have enough time to address smoking cessation with my clients. |  |  |  |  |  |
| Among former drug users, quitting smoking may increase their chances of relapsing back to drug use. |  |  |  |  |  |
| Among my patients, addiction to nicotine is a less-urgent problem than addiction to alcohol and other drugs. |  |  |  |  |  |
| The costs associated with tobacco cessation medications and counseling are barriers to treatment for my clients. |  |  |  |  |  |

**Tobacco Use Assessment and Cessation Advice (2-2)**

| **PLEASE INDICATE WHICH OF THE FOLLOWING METHODS YOU REGULARLY INCORPORATE TO HELP YOUR HIV INFECTED PATIENTS QUIT SMOKING:**  **(CHECK ALL THAT APPLY)** | | |
| --- | --- | --- |
|  | Utilize an electronic medical record reminder to ask all patients about their tobacco use.  Prescribe nicotine replacement therapy (NRT) (such as the nicotine patch or spray).  Provide free samples of NRT or medications to patients.  Schedule a follow-up phone call or visit with patients who express intentions to quit smoking. | Refer clients to a local community smoking cessation program.  Refer clients to 1-800-QUIT-NOW  Provide readily accessible educational materials about smoking cessation in waiting and exam rooms.  None of the above |

**Opinion (1-1)**

| **FINALLY, WE WOULD LIKE TO PROVIDE AN OPPORTUNITY FOR YOU TO COMMENT.**  **THIS SECTION IS STRICTLY OPTIONAL.** | |
| --- | --- |
| In your opinion, what are the most important barriers to improving access to appropriate HIV care?  (optional) |  |
| Do you have any additional comments or feedback?  (optional) |  |
